# Bortezomib, lenalidomide and dexamethasone (VRd) vs carfilzomib, lenalidomide and dexamethasone (KRd) as induction therapy in newly diagnosed multiple myeloma

**DOI:** 10.1038/s41408-023-00882-y

**Published:** 2023-07-25

**Authors:** Carlyn Rose Tan, Andriy Derkach, David Nemirovsky, Amanda Ciardiello, Benjamin Diamond, Malin Hultcrantz, Hani Hassoun, Sham Mailankody, Urvi Shah, Kylee Maclachlan, Dhwani Patel, Oscar B. Lahoud, Heather J. Landau, David J. Chung, Gunjan L. Shah, Michael Scordo, Sergio A. Giralt, Alexander Lesokhin, Saad Z. Usmani, Ola Landgren, Neha Korde

**Affiliations:** 1 Myeloma Service, Department of Medicine, Memorial Sloan Kettering Cancer Center, New York, NY USA; 2 Department of Epidemiology and Biostatistics, Memorial Sloan Kettering Cancer Center, New York, NY USA; 3 Myeloma Division, Sylvester Comprehensive Cancer Center, University of Miami, Miami, FL USA; 4 Adult Bone Marrow Transplant Service, Department of Medicine, Memorial Sloan Kettering Cancer Center, New York, NY USA

**Keywords:** Myeloma, Combination drug therapy

## Abstract

Lenalidomide and dexamethasone with bortezomib (VRd) or carfilzomib (KRd) are commonly used induction regimens in the U.S. This single-center, retrospective study evaluated outcomes and safety of VRd and KRd. Primary endpoint was progression-free survival (PFS). Of 389 patients with newly diagnosed multiple myeloma, 198 received VRd and 191 received KRd. Median PFS was not reached (NR) in both groups; 5-year PFS was 56% (95%CI, 48–64%) for VRd and 67% (60–75%) for KRd (*P* = 0.027). Estimated 5-year EFS was 34% (95%CI, 27–42%) for VRd and 52% (45–60%) for KRd (*P* < 0.001) with corresponding 5-year OS of 80% (95%CI, 75–87%) and 90% (85–95%), respectively (*P* = 0.053). For standard-risk patients, 5-year PFS was 68% (95%CI, 60–78%) for VRd and 75% (65–85%) for KRd (*P* = 0.20) with 5-year OS of 87% (95%CI, 81–94%) and 93% (87–99%), respectively (*P* = 0.13). For high-risk patients, median PFS was 41 months (95%CI, 32.8–61.1) for VRd and 70.9 months (58.2-NR) for KRd (*P* = 0.016). Respective 5-year PFS and OS were 35% (95%CI, 24–51%) and 69% (58–82%) for VRd and 58% (47–71%) and 88% (80–97%, *P* = 0.044) for KRd. Overall, KRd resulted in improved PFS and EFS with a trend toward improved OS compared to VRd with associations primarily driven by improvements in outcome for high-risk patients.

## Introduction

Multiple myeloma (MM) is the second most common hematologic malignancy with over 35,000 new individuals being affected annually in the United States and global incidence increasing sharply in recent decades [1, 2]. Over the past two decades, the therapeutic landscape and management of MM has evolved substantially, reflected in median overall survival (OS) of more than 10 years for newly diagnosed patients in select subgroups. Currently, there is no established curative treatment for multiple myeloma.

The use of proteasome inhibitors (PIs) and immunomodulatory drugs (IMiDs) in patients with newly diagnosed multiple myeloma (NDMM) has improved clinical outcomes, independent of transplant eligibility [3–7]. Initially, bortezomib, lenalidomide, and dexamethasone (VRd) was developed as an induction regimen for patients with NDMM based on results from a phase 1/2 trial [8] and subsequently found to result in improved progression-free survival (PFS) and OS with an acceptable safety profile [9–12]. However, the high incidence of bortezomib-induced peripheral neuropathy, which is often irreversible, can result in significant morbidity and prevent its long-term use, despite use of the subcutaneous formulation or alteration in the dosing schedule. Carfilzomib, a next-generation PI, in combination with lenalidomide and dexamethasone (KRd) has been demonstrated in multiple phase 2 studies in patients with NDMM to have high overall response rates (ORR) and deep, durable responses with tolerable side effects [13–16]. Rare but serious side effects associated with carfilzomib include cardiovascular events. Currently only one randomized study (ENDURANCE trial) has compared VRd and KRd in patients with NDMM [17]. However, patients with high-risk cytogenetics and those intended for immediate transplant were excluded because a parallel randomized phase 2 study, SWOG 1211, was concurrently enrolling patients with high-risk MM [18]. In standard-risk NDMM, the ENDURANCE trial demonstrated no differences in PFS or OS between VRd and KRd. Single-center retrospective studies have also compared VRd and KRd regimens in high-risk MM [19].

Although the overall toxicity profiles of VRd and KRd are considered favorable, their prolonged use warrants a heightened vigilance for toxicity evaluation, and treating physicians need to carefully balance efficacy and toxicity profiles for each patient. Since patients enrolled on clinical trials, per eligibility criteria, are less frail and have fewer comorbidities than patients in the general population [20, 21], we were motivated to conduct a retrospective study to define the efficacy and safety of VRd and KRd in patients with standard and high-risk MM.

## Methods

Using an in-house data query platform, DataLine, we retrospectively identified patients with NDMM treated with VRd and KRd at Memorial Sloan Kettering Cancer Center (MSK) between January 1, 2015 and December 31, 2019. The last follow-up date was September 30, 2022. This study received approval from the Institutional Review Board at MSK. The research was performed in compliance with the terms from the Declaration of Helsinki and was waived from the obligation to obtain written informed consent. Data underwent peer-based quality check for completeness and internal consistencies. Patients with NDMM were included if they completed at least 1 cycle of VRd or KRd as induction therapy. Patients were also included if they received ≤1 cycle of a different induction regimen prior to receiving VRd or KRd. The decision regarding which induction regimen a patient received was based on the treating physician’s discretion and patient preferences. Patients were evaluated for autologous stem cell transplant (ASCT) based on institutional guidelines and proceeded with early or deferred ASCT based on patient and physician preference. High-risk FISH/SNP signature was defined as one or more of the following abnormalities: 1q + , t(4;14), t(14;16), t(14;20), and del(17p).

Response was assessed using the International Myeloma Working Group (IMWG) consensus criteria for response [22]. Minimal residual disease (MRD) testing, if completed, was performed during or after completion of induction cycles using a validated flow cytometry assay with at least a limit of detection of 10^−5^ [23].

Patients’ charts in the electronic health records (EHR) were reviewed to evaluate for specific adverse events (AEs), including pulmonary and cardiovascular events, hypertension (worsening from baseline or requiring medication modifications), renal complications, and peripheral motor or sensory neuropathy. AEs were graded using Common Terminology Criteria for Adverse Events (CTCAE), version 5.0 retrospectively. AE data was consolidated for each patient and AE category by taking the worst grade across time and category (pulmonary and cardiovascular events, hypertension, renal events, and peripheral motor or sensory neuropathy) and counting patients documented to have multiple AEs within a category only once. All available data were reviewed to assess reversibility defined as complete resolution or return to baseline of toxicities up to 6 months following completion of induction therapy.

### Statistical analysis

Descriptive statistics was used to summarize patient and disease characteristics and toxicities experienced across the VRd and KRd groups. These variables were compared between the two treatment groups using Fisher’s exact test for categorical variables and Wilcoxon rank-sum tests for continuous variables. Event-free survival (EFS) was defined as the time from start of induction therapy to death, progression of disease and change in the line of therapy following initial VRd or KRd induction, whichever came first. Progression-free survival (PFS) was defined as the time from the start of induction therapy until progression of disease or death from any cause. Overall survival (OS) was defined as the time from start of induction therapy to death from any cause. Left truncation was used in patients who transferred and completed their induction treatment to MSK. EFS, PFS, and OS were estimated by the Kaplan-Meier method, and differences in time to event outcome between VRd and KRd were assessed by log-rank test. Median follow-up was calculated using reverse Kaplan-Meier method. Multivariable Cox proportional hazard regression models were used to estimate the hazard ratios (HR) and 95% confidence intervals (CIs) for the comparison of effects of induction regimens on risk of time to event outcomes, adjusting for age at time of induction start (years, continuous), cytogenetic risk (standard, high), R-ISS Stage (I, II, and III), cardiac history (see Table 1 for list of medical conditions included) and time-dependent covariates, including early autologous stem cell transplant, more than 6 cycles of induction therapy and maintenance. Proportional hazards test based on weighted residuals was used to check for violations of the assumptions of Cox proportional-hazard regression [24]. Since the assumptions were violated in the OS analysis, two separate Cox proportional-hazard regression were fitted to: (1) all patients with follow-up time truncated at 5.5 years and (2) patients with follow-up time longer than 5.5 years (e.g. landmark analysis at 5.5 years). Different follow-up thresholds ranging from 5 to 6 years were checked in sensitivity analysis to qualitatively confirm the main findings. To confirm our findings, survival analysis with propensity weighting was additionally performed. We constructed propensity weights using logistic regression with the same set of covariates except for age at diagnosis, which was modeled by cubic splines. Variables with *P* values < 0.05 were considered statistically significant. All analyses were performed using CRAN R Version 3.3.0 (The R Foundation for Statistical Computing, Vienna, Austria).

**Table 1:** Baseline patient characteristics.

Characteristics	VRd (*N* = 198)	KRd (*N* = 191)	*P* value
Median age—yr (IQR)	65 (57–72)	62 (55–68)	0.006
Age category—n (%)			
≤70 yr	137 (69)	154 (80)	
>70 yr	61 (31)	37 (20)	
Male sex (%)	102 (52)	106 (55)	0.4
Race, n (%)			0.6
White	148 (75)	132 (69)	
Black	25 (13)	26 (14)	
Asian	7 (4)	10 (5)	
Other/unknown	18 (9)	23 (12)	
ECOG Performance Status - n/total n (%)			0.3
0	67/150 (45)	72/135 (53)	
1	66/150 (44)	50/135 (37)	
≥2	17/150 (11)	13/135 (10)	
Creatinine Clearance >60mL/min—n (%)	153 (77)	162 (85)	0.28
History of HTN - n (%)	98 (49)	91 (48)	0.7
Cardiac history* - n (%)	45 (23)	24 (13)	0.008
History of Peripheral Neuropathy—n (%)	34 (17)	36 (19)	0.69
Stage (R-ISS) - n/total n (%)			0.43
I	68/178 (38)	76/171 (44)	
II	101/178 (57)	89/171 (52)	
III	9/178 (5)	6/171 (4)	
Missing	20	20	
Cytogenetic risk—n/total n (%)			0.021
High-risk^†^	67/182 (37)	87/178 (49)	
Standard-risk	115/182 (63)	91/178 (51)	
Missing	16	13	
del(17p)—n/total n (%)	13/182 (7)	25/178 (14)	0.03
t(4;14)—n/total n (%)	8/182 (4)	15/178 (8)	0.11
t(14;16)—n/total n (%)	3/182 (2)	7/178 (4)	0.2
t(14;20)—n/total n (%)	2/182 (1)	3/178 (2)	0.7
Gain/amp 1q—n/total n (%)	58/182 (32)	64/178 (36)	0.4
Number of high-risk cytogenetic abnormalities—n/total n (%)			0.6
1	51/67 (76)	63/87 (72)	
≥2	16/67 (24)	24/87 (28)	
Early ASCT^^^—n (%)	93 (47)	88 (46)	0.90
Maintenance			<0.001
Yes—n/total n (%)	138/194 (71)	171/189 (90)	
Lenalidomide—n/total n (%)	125/138 (91)	154/171 (90)	0.6
IMiD/PI—n/total n (%)	4/138 (3)	7/171 (4)	
Other—n/total n (%)	9/138 (6)	10/171 (6)	
No^#^ - n/total n (%)	56/194 (29)	18/189 (10)	

*Cardiac history include atrial fibrillation, atrial flutter, coronary artery disease, right bundle branch block, valvular heart disease, aortic aneurysm, sick sinus syndrome, systolic heart failure, non-ischemic cardiomyopathy, heart failure with preserved ejection fraction, heart block ^†^high risk include 1q+, t(4;14), t(14;16), t(14;20), del(17p). ^^^Early ASCT defined as ASCT within 12 months of induction therapy start without progression of disease. ^#^Includes patients who switched therapies without PD, switched therapies because of PD, died, or refused maintenance therapy.

*ECOG* Eastern Cooperative Oncology Group performance, *ASCT* autologous stem cell transplant, *IMiD* immunomodulatory agent, *PI* proteasome inhibitor.

## Results

Between January 1, 2015 and December 31, 2019, 389 NDMM patients received VRd (*n* = 198) and KRd (*n* = 191) induction at MSK. Baseline characteristics are summarized in Table 1. Patients with high-risk cytogenetic abnormalities (HRCA) were more likely to receive KRd (37% vs 49%; *P* = 0.021), and conversely, patients with known cardiovascular disease were more likely to receive VRd (23% vs 13%; *P* < 0.01). The median age at therapy initiation was 65 (interquartile range, IQR, 57–72) in the VRd group and 62 (IQR, 55–68) in the KRd group (*P* = 0.006). The median number of cycles was 6 (IQR, 4–7) for VRd and 6 (IQR, 5–8) for KRd. During induction, 147 (74%) VRd-treated and 167 (87%) KRd-treated patients collected autologous hematopoietic cells; the success rate for collection of hematopoietic cells was greater than 90% in both groups. Following induction therapy, 47% of patients in the VRd group and 46% of patients in the KRd group underwent high-dose chemotherapy followed by ASCT. In the VRd-treated patients, 71% received maintenance therapy post-transplant or after induction for patients who deferred early ASCT while 90% of KRd-treated patients proceeded to maintenance therapy. Among the patients who received maintenance therapy, 91% in the VRd group and 90% in the KRd group received lenalidomide maintenance.

### Efficacy

#### Response to therapy

The overall response rate at the end of induction was 91% and 99% for VRd and KRd, respectively (*P* < 0.01) (Table 2). There were deeper responses associated with KRd compared to VRd. The ≥complete response (CR) and ≥very good partial response (VGPR) rates for VRd vs KRd, respectively, were 25% vs 41% (*P* < 0.01) and 63% vs 86% (*P* < 0.01). Among patients who were evaluable for MRD status post-induction, MRD negativity was 27% (30/113) with VRd and 40% (69/174) with KRd (*P* = 0.02). Based on small numbers, the MRD negativity rate for evaluable patients with standard-risk was 29% (20/69) and 46% (40/87) with VRd and KRd, respectively (*P* = 0.03). For high-risk patients, the corresponding numbers were 21% (8/39) and 29% (23/78) (*P* = 0.30).

**Table 2:** Treatment and responses.

	VRd (*N* = 198)	KRd (*N* = 191)	*P* value
Number of cycles—n (%)			
1-3	34 (17)	12 (6)	<0.01
4–5	56 (28)	36 (19)	0.02
6-8	90 (45)	114 (60)	<0.01
≥9	18 (9)	29 (15)	0.09
Best overall response to induction—n (%)			<0.01
sCR/CR	49 (25)	79 (41)	<0.01
VGPR	75 (38)	86 (45)	0.12
PR	57 (29)	24 (13)	<0.01
MR	7 (4)	2 (1)	0.18
SD	8 (4)	0 (0)	<0.01
PD	2 (1)	0 (0)	0.25
Minimal residual disease status—n (%)	VRd (*N* = 113)	KRd (*N* = 174)	
Negative status*	30 (27)	69 (40)	0.022

*During or after completing induction therapy.

#### Progression-free survival, event-free survival, and overall survival for all patients

##### PFS

**Fig. 1:**
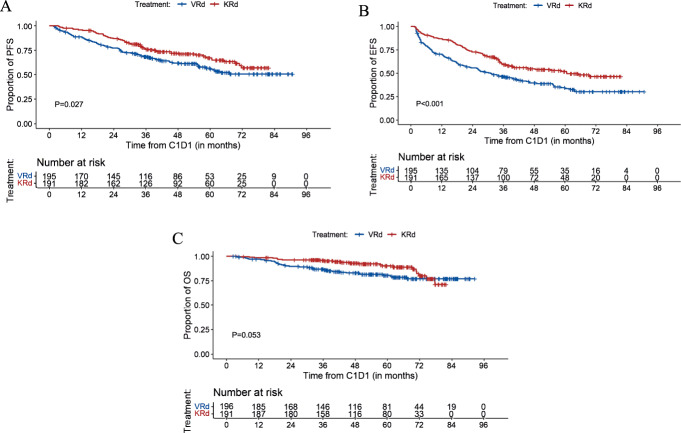
Progression-free survival, event free survival, and overall survival. **A** Progression-free survival (PFS); (**B**) Event free survival (EFS); (**C**) Overall survival (OS).

The median follow-up for the overall population was 58.8 months (95%CI, 55–62.5) with 59.8 months for the VRd group and 57.5 months for KRd. Median PFS was not reached (95%CI, 58.4-NR) in the VRd group and not reached in the KRd group (95%CI, 71.7-NR). Estimated 5-year PFS was 56% (95%CI, 48–64%) for VRd and 67% (95%CI, 60–75%) for KRd (*P* = 0.027) (Fig. 1A). Since baseline characteristics of the two groups were not matched, propensity score weighting was performed and demonstrated a statistically significant benefit in PFS associated with KRd (Supplementary Table 1). In a separate PFS sensitivity analysis, 181 (93 VRd and 88 KRd) patients who received early ASCT, defined as ASCT completed within 12 months of induction start date without progressive disease, were censored at the time of transplant. In this analysis, PFS was significantly longer in the KRd group (*P* = 0.034) with 5-year PFS rates of 52% (95%CI, 42–65%) for VRd-treated patients and 65% (95%CI, 56–76%) for KRd (Supplementary Fig. 1).

##### EFS

Reflective of standard clinical practice in the United States, there were patients who changed therapy in the absence of standard definition of progressive disease per IMWG criteria. To account for such potential bias, we estimated EFS and found the 5-year rates were 34% (95%CI, 27–42%) and 52% (95%CI, 45–60%) in the VRd and KRd groups, respectively (*P* < 0.001) (Fig. 1B).

##### OS

At the median follow-up of 51.6 months, 37 patients died in the VRd group and 22 died in the KRd group. During induction, 2 patients died while receiving VRd (one patient died from a hemorrhagic stroke and one patient from unknown cause during cycle 4). No patients died during KRd induction. Median OS has not been reached in either group. The Kaplan-Meier estimates for OS at 5 years were 80% (95%CI 75–87%) in the VRd group and 90% (85–95%) in the KRd group (*P* = 0.053) (Fig. 1C).

After 5 years of follow-up, which included the coronavirus disease 2019 (COVID-19) pandemic, 3 patients in the VRd group and 7 patients in the KRd group died. In the VRd-treated patients, 1 patient died from a secondary primary malignancy and 2 patients died from progressive disease (plasma cell leukemia). In the KRd group, 2 patients died from progressive disease (PD), 1 patient died from infection, 1 patient had PD and disseminated fungal infection, and 3 patients had unknown cause of death. Since 7 out of the 22 deaths in the KRd group were observed after 5 years of follow-up, the main assumption of the Cox proportional-hazards model was not satisfied (*P* = 0.002). We accounted for observing an inconstant effect of induction over time by reporting an analysis of OS for all patients with follow-up truncated at 5.5 years and a landmark analysis of OS at 5.5 years. When follow-up was truncated at 5.5 years, KRd was associated with improved OS (*P* = 0.004); in contrast, for landmark analysis at 5.5 years, VRd was associated with longer OS (*P* = 0.019) (Supplementary Fig. 2A, B).

#### PFS, EFS, and OS of subgroups based on cytogenetic risk

Cytogenetic data was available for 182 (92%) and 178 (93%) patients treated with VRd and KRd induction, respectively. HRCAs were identified in 67 (37%) VRd-treated patients and 87 (49%) KRd-treated patients. In the VRd group, 16 (9%) had ≥2 HRCAs compared to 24 (13%) in the KRd group.

##### PFS

**Fig. 2:**
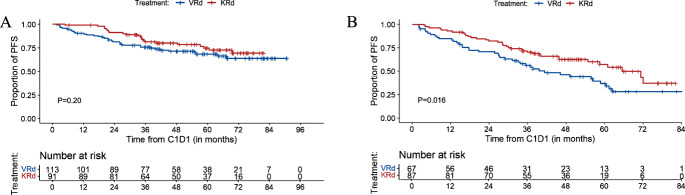
PFS for patients with standard-risk and high-risk cytogenetics. **A** PFS for patients with standard-risk cytogenetics; (**B**) PFS for patients with high-risk cytogenetics.

The median PFS for patients with standard-risk cytogenetics was not reached in both groups (*P* = 0.20) (Fig. 2A). The estimated 5-year PFS was 68% (95%CI, 60%-78%) for VRd and 75% (65–85%) for KRd. For patients with HRCA, the median PFS was 41 months (95%CI, 32.8–61.1) for VRd and 70.9 months (95%CI, 58.2-NR) for the KRd group (*P* = 0.016) with estimated 5-year PFS rate of 35% (95%CI, 24–51%) and 58% (47–71%), respectively (Fig. 2B). For the patients with gain/amp 1q (VRd 58 and KRd 64), there was a trend toward improved PFS associated with KRd compared to VRd with 5-year PFS estimates of 62% (95%CI, 50–77%) and 40% (95%CI, 27–58%), respectively (*P* = 0.051) (Supplementary Fig. 3).

##### EFS

For patients with standard-risk cytogenetics, median EFS was 41.7 months (95%CI, 26.2-NR) for VRd and NR (95%CI, NR-NR) for KRd with 5-year estimated EFS of 44% (95%CI, 35–55%) and 60% (95%CI, 50–71%), respectively (*P* = 0.007) (Supplementary Fig. 4A). In high-risk patients, median EFS was 26.6 months (95%CI, 15.4–41) for VRd and 39.3 months (95%CI, 29.8-NR) for KRd with 5-year EFS of 20% (95%CI, 11–35%) and 43% (95%CI, 33–56%), respectively (*P* = 0.016) (Supplementary Fig. 4B).

##### OS

**Fig. 3:**
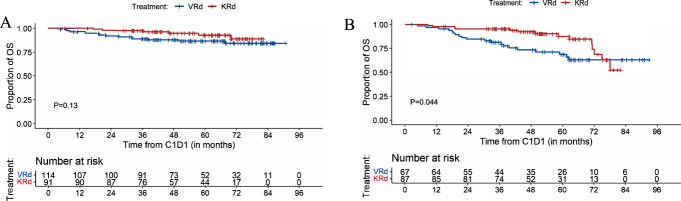
OS for patients with standard-risk and high-risk cytogenetics. **A** OS for patients with standard-risk cytogenetics; (**B**) OS for patients with high-risk cytogenetics.

Estimated 5-year OS for patients with standard-risk cytogenetics was 87% (95%CI, 81%-94%) for VRd-treated patients and 93% (87–99%) for KRd (*P* = 0.13) (Fig. 3A). Among high-risk patients, the 5-year OS rate for VRd and KRd induction was 69% (95%CI, 58–82%) and 88% (80–97%), respectively (*P* = 0.044) (Fig. 3B). Similar to the main analysis of OS, we analyzed OS for these subgroups based on follow-up period (truncated at 5.5 years and landmark point at 5.5 years). For standard-risk patients, there were no statistically significant differences in OS comparing VRd and KRd in both analyses (all patients with follow-up truncated at 5.5 years: *P* = 0.10 and landmark analysis at 5.5 years: *P* = 0.80) (Supplementary Fig. 5A, B). For high-risk patients, KRd compared to VRd was associated with improved OS when follow-up for all patients was truncated at 5.5 years (*P* = 0.002); for the small group of patients used in the landmark analysis at 5.5 years VRd was associated with longer OS than KRd (*P* = 0.04) (Supplementary Fig. 6A, B).

#### Multivariable analysis

We conducted a multivariate analysis for important clinical variables that may affect survival outcomes (Table 3). Multivariable analysis for PFS showed that KRd induction (HR 0.69, 95%CI 0.48–0.98; *P* = 0.039), standard-risk cytogenetics (HR 2.20, 95%CI 1.52–3.17; *P* < 0.001), R-ISS Stage I vs II (HR 1.51, 95%CI 1.02–2.22; *P* = 0.037), and R-ISS Stage I vs III (HR 2.28, 95%CI 1.08–4.81; *P* = 0.037) were associated with improved PFS (Table 3 and Supplementary Table 4).

**Table 3:** Multivariable analysis for all groups: PFS and OS.

	PFS		OS*	
Variable	Hazard ratio (95%CI)	*P* value	Hazard ratio (95%CI)	*P* value
Induction		0.039		0.01
VRd	1		1	
KRd	0.69 (0.48–0.98)		0.44 (0.23–0.84)	
Gender		>0.9		>0.9
Female	1		1	
Male	1.01 (0.71–1.44)		1.02 (0.56–1.88)	
Age	1.00 (0.99–1.02)	0.6	1.03 (1.0–1.06)	0.1
Cardiac History		0.2		>0.9
Yes	1		1	
No	0.74 (0.47–1.15)		1.00 (0.47–2.13)	
Cytogenetic risk		<0.001		0.024
Standard	1		1	
High	2.20 (1.52–3.17)		2.00 (1.09–3.66)	
R-ISS Stage		0.037		0.14
I	1		1	
II	1.51 (1.02–2.22)		1.34 (0.68–2.63)	
III	2.28 (1.08–4.81)		3.25 (1.09–9.69)	
Early ASCT		0.8		0.024
No	1		1	
Yes	0.95 (0.59–1.50)		0.43 (0.21–0.91)	
Number of cycles > 6	1.18 (0.73–1.88)	0.5	0.73 (0.36–1.45)	0.4

*Multivariate analysis for OS includes patients with follow-up truncated at 5.5 years. Results from landmark analysis at 5.5 years available in Data Supplement.

Multivariable analysis for OS with follow-up truncated at 5.5 years confirmed that KRd was associated with improved OS (HR 0.44, 95%CI 0.23–0.84; *P* = 0.01) after adjusting for important clinical variables (Table 3). Additionally, other variables associated with improved OS included standard-risk cytogenetics (HR 2.00, 95%CI 1.09–3.66; *P* = 0.024) and early ASCT (HR 0.43, 95%CI 0.21–0.91; *P* = 0.024). In the landmark analysis at 5.5 years, association with KRd and longer OS was no longer statistically significant (HR 9.88, 95%CI 0.88–111; *P* = 0.063) after adjusting for the same confounders (Supplementary Table 5A).

VRd or KRd induction was not associated with improvement in PFS and OS in NDMM patients with standard-risk cytogenetic abnormalities (Table 4A). In the cohort of standard-risk patients with follow-up truncated at 5.5 years, age was a significant variable associated with OS (HR 1.08, 95%CI 1.02–1.15; *P* = 0.004). Within the HRCA subgroup, multivariable analysis demonstrated that KRd induction (HR 0.59, 95%CI 0.37–0.95; *P* = 0.029), no known significant cardiac history prior to start of induction (HR 0.49, 95%CI 0.27–0.89; *P* = 0.024), R-ISS Stage I vs II (HR 2.31, 95%CI 1.26–4.24; *P* = 0.008), and R-ISS Stage I vs III (HR 3.16, 95%CI 1.21–8.24; *P* = 0.008) were associated with improved PFS (Table 4B). In HRCA patients with follow-up truncated at 5.5 years, KRd (HR 0.37, 95%CI 0.16–0.85; *P* = 0.016) and early ASCT (HR 0.34, 95%CI 0.14–0.85; *P* = 0.019) were associated with improved OS. For patients with HRCA and >5.5 years of follow-up, the association between the use of VRd or KRd with improvement in OS was not estimable (Supplementary Table 5C).

**Table 4:** A. Multivariable analysis for standard-risk subgroup: PFS and OS. B. Multivariable analysis for high-risk subgroup: PFS and OS.

	PFS		OS*	
Variable	Hazard ratio (95%CI)	*P* value	Hazard ratio (95%CI)	*P* value
**A**				
Induction		0.2		0.1
VRd	1		1	
KRd	0.67 (0.38–1.19)		0.42 (0.14–1.22)	
Gender		>0.9		0.8
Female	1		1	
Male	1.03 (0.60–1.78)		0.90 (0.35–2.31)	
Age	1.01 (0.99–1.04)	0.3	1.08 (1.02–1.15)	0.004
Cardiac history		0.8		0.5
Yes	1		1	
No	1.13 (0.54–2.37)		1.51 (0.42–5.44)	
R-ISS stage		0.8		0.5
I	1		1	
II	1.10 (0.63–1.92)		0.70 (0.26–1.89)	
III	1.60 (0.36–7.15)		1.86 (0.31–11.4)	
Early ASCT		0.3		0.4
No	1		1	
Yes	0.70 (0.36–1.36)		0.61 (0.16–2.26)	
Number of cycles >6	1.69 (0.84–3.42)	0.14	1.48 (0.48–4.55)	0.5
**B**				
Induction		0.029		0.016
VRd	1		1	
KRd	0.59 (0.37–0.95)		0.37 (0.16–0.85)	
Gender		0.6		0.7
Female	1		1	
Male	0.88 (0.54–1.45)		1.19 (0.51–2.75)	
Age	0.99 (0.97–1.01)	0.6	1.00 (0.96–1.04)	>0.9
Cardiac history		0.024		0.7
Yes	1		1	
No	0.49 (0.27–0.89)		0.83 (0.31–2.17)	
R-ISS stage		0.008		0.11
I	1		1	
II	2.31 (1.26–4.24)		2.43 (0.82–7.15)	
III	3.16 (1.21–8.24)		4.25 (0.92–19.7)	
Early ASCT		0.6		0.019
No	1		1	
Yes	0.85 (0.46–1.59)		0.34 (0.14–0.85)	
Number of cycles > 6	0.91 (0.47–1.78)	0.8	0.41 (0.15–1.13)	0.074

*Multivariate analysis for OS includes patients with follow-up truncated at 5.5 years. Results from landmark analysis at 5.5 years available in Data Supplement.

### Safety

Overall (including all grades), 15% of patients developed pulmonary and cardiovascular events and 30% developed new or worsening neuropathy during therapy (Supplementary Table 2). Patterns of these toxicities are described in further detail below.

#### Cardiovascular and pulmonary adverse events

In the VRd group, 5% of patients experienced at least one grade (G) 2 or higher cardiovascular and pulmonary AE compared to 8% in the KRd group; these events were reversible in 67% and 88% of patients who experienced a grade 2 or higher event while receiving VRd and KRd, respectively. The incidence of grade ≥2 pulmonary and cardiovascular AEs are summarized in Supplementary Table 3.

#### Peripheral neuropathy

For patients who received VRd as their frontline regimen, 30 (15%) patients had sensory alteration or paresthesia interfering with function and/or symptomatic weakness interfering with function, or more severe forms of neuropathy (grade ≥2). Three (2%) patients had improvement to baseline or resolution of symptoms within 6 months of completing therapy, and 27 (14%) developed chronic neuropathy. VRd-treated patients who developed peripheral neuropathy required various interventions, including opiates (*N* = 16), pregabalin/gabapentin/duloxetine (*N* = 35), rehabilitation/physical therapy (*N* = 4), assistive devices for ambulation such as canes, walkers, and wheelchairs (*N* = 5). None of the patients treated with KRd developed grade ≥2 peripheral neuropathy.

#### Adverse events and treatment discontinuation

Overall, 10% and 7% of patients treated with VRd and KRd, respectively, required treatment discontinuation due to AEs. Peripheral neuropathy resulted in treatment discontinuation for 9% of patients treated with VRd. Cardiovascular and pulmonary AEs (myocardial infarction G3, *N* = 1; pulmonary hypertension G2, *N* = 2; pulmonary hypertension G1, *N* = 4; pericarditis G2, *N* = 1; dyspnea G2, *N* = 1; dyspnea G1, *N* = 1; chest pain G1, *N* = 1; atrial fibrillation G3, *N* = 1) resulted in treatment discontinuation for 6% of KRd-treated patients.

## Discussion

We conducted a real-world study with 389 patients with NDMM prospectively treated with VRd or KRd from a single institution during a 5-year period. Our primary aims were to investigate measures of efficacy, including depth of response, PFS, EFS, and OS, and characterize the safety profile of these two regimens in standard and high-risk patients. Overall, we found better outcomes associated with KRd compared to VRd, including depth of response with patients achieving ≥CR rate (25% vs. 41%, *P* < 0.01), 5-year PFS rates (56% vs. 67%, *P* = 0.027), and 5-year EFS rates (34% vs. 52%, *P* < 0.001) with the VRd and KRd groups, respectively. There was a trend toward improved 5-year OS rates associated with KRd (80% vs. 90%, *P* = 0.053). Of note, our KRd cohort was enriched for high-risk MM patients, but propensity weight scoring analysis still confirmed KRd benefit in the PFS evaluation. Additionally, KRd demonstrated PFS improvement over VRd, regardless of early ASCT status. In the ENDURANCE trial, the median PFS was 34.4 and 34.6 months for VRd and KRd-treated standard-risk patients, respectively [17]. Other studies have reported findings suggesting that VRd and KRd induction can achieve better results than findings from the ENDURANCE trial. Our results, demonstrating median PFS for VRd and KRd were not reached after a median follow-up of 58.8 months, differ from ENDURANCE, but are consistent with other VRd (DETERMINATION, RVD1000) and KRd (MMRC, FORTE) datasets [12, 13, 25, 26].

In this real-world study, we also found a high proportion of patients changing therapy in the absence of progressive disease per IMWG criteria. To overcome issues of statistical bias, we performed EFS sensitivity analysis and found that KRd was associated with significantly improved EFS compared to VRd after adjusting for age, cytogenetic risk, R-ISS stage, and early ASCT. Importantly, clinical trials allowing patients to change therapy without meeting IMWG criteria for progression should report EFS and time to treatment failure in the main analysis in order to properly overcome the influence of censoring bias, which inherently will occur in PFS analysis [27]. It seems reasonable to conjecture that increased access to more sensitive testing (for example, blood-based MRD testing) will increase the proportion of patients changing therapy in the absence of progressive disease by IMWG criteria in the future.

On subgroup analysis, we did not detect a significant improvement in PFS associated with KRd induction compared to VRd in patients with standard-risk cytogenetics. Median PFS was not reached in both VRd and KRd groups (*P* = 0.20). The ENDURANCE trial, which was conducted only in standard-risk patients also did not detect PFS differences between VRd and KRd regimens. Conversely, our real-world study shows a clear PFS benefit associated with KRd compared to VRd in patients with HRCA demonstrating median PFS of 41 months vs. 70.9 months in VRd and KRd groups, respectively (*P* = 0.016). Compared to other studies, our study is consistent with PFS rates reported in high-risk multiple myeloma patients receiving either VRd or KRd, including RVD1000, SWOGS1211, and FORTE [12, 18, 26]. Importantly, these trends for KRd benefit over VRd were seen in both PFS and OS (truncated at 5.5 years of follow-up) multivariate analysis for high-risk and overall group but not seen in standard-risk multivariate analysis.

In addition, there was better tolerability with KRd compared to VRd, reflected in an absence of severe neuropathy and substantially lower rates of cardiovascular and thromboembolic events compared to select prior studies [17, 28, 29]. In our study, 16% of the patients treated with VRd developed grade ≥2 peripheral neuropathy with 14% having persistent symptoms more than 6 months after completion of induction. Our findings are an important reflection of clinically impactful bortezomib-induced peripheral neuropathy since the most common reason for treatment-discontinuation due to AEs in the VRd group was peripheral neuropathy. In contrast, we did not find any grade ≥2 peripheral neuropathy with KRd, and none of the patients required neuropathy-specific interventions. Carfilzomib is known to have a cardiovascular signal. In our study, we captured patients who experienced grade ≥2 cardiovascular and pulmonary AE (VRd 5% vs KRd 8%). These events were reversible in the majority of patients (VRd 66% vs KRd 87%). The lower rates of cardiopulmonary AEs associated with KRd in our study is likely driven by optimized intravenous fluid management and modern anticoagulation therapy given at our institution [30]. The ENDURANCE trial reported a composite of treatment-related grade ≥3 cardiac and pulmonary disorders occurring in 16% of KRd-treated patients [17, 30], while several other clinical trials have not found significantly elevated rates of grade 3 or higher pulmonary and cardiovascular AEs associated with carfilzomib, ranging from 2%-5% in the FORTE and MMRC trials [13, 26, 31–33].

Strengths of the study include large sample size and uniform treatment administration and supportive therapies at a high-volume myeloma program in the United States during a 5-year period. Our real-world study design allowed for inclusion of patients with various comorbidities reflective of the general population, who are frailer than those treated on clinical trials. We acknowledge that because of these factors, there are varying treatment schedules and doses given in the real-world setting to mitigate side effects and personalize treatment among the included patients. Weaknesses include retrospective study design without randomization leading to inherent selection bias. To overcome inherent biases, we adjusted and stratified for confounders. However, while a Cox-regression analysis was used to adjust for a heterogenous patient population and propensity score weighting was performed, it is likely that patient heterogeneity and treating physician bias had some uncompensated impact on the analysis. When the proportional-hazards assumption in the Cox regression model was not met, truncated analysis at change point of 5.5 years of follow-up was performed. There were only a small group of patients (61 VRd and 54 KRd) with longer than 5.5 years of follow-up in both groups, making it difficult to draw any conclusions. We failed to see a difference in overall survival between VRd and KRd in the landmark analysis at 5.5 years after adjusting for confounding variables, and longer follow-up is needed. Moreover, given the retrospective nature of the study, AEs were not prospectively collected with the same rigor as in clinical trials and only AEs documented in the EMR were captured for analysis.

In summary, this real-world data analysis involving almost 400 patients with NDMM – including both standard-risk and high-risk patients – from a high-volume treatment center treated with VRd or KRd combination therapy provide clinically important information for treating physicians and patients with newly diagnosed multiple myeloma. Future studies are needed to investigate the role of added monoclonal antibodies to these combination therapies and to investigate the role of MRD testing for clinical decision making.

## Supplementary information

Data Supplement
