# Supplementary material for: Bortezomib, lenalidomide and dexamethasone (VRd) vs carfilzomib, lenalidomide and dexamethasone (KRd) as induction therapy in newly diagnosed multiple myeloma
Source: Blood Cancer J. 2023 Jul 25;13(1):112. doi: 10.1038/s41408-023-00882-y (PMC10368661; doi:10.1038/s41408-023-00882-y)

Data Supplement

Table 1. Propensity Scoring

Using age, cytogenetics, R-ISS Stage, and cardiac history

	PFS	
**Variable**	Hazard Ratio (95% CI)	P value
Induction		0.03
VRd	1	
KRd	0.52 (0.29-0.92)	

Table 2. Adverse events during induction therapy

Event – no (%)	VRd (n=198)	KRd (n=191)	P-value
**Pulmonary and cardiovascular AEs**	17 (9)	41 (21)	<0.01
**Reversed**	14/17 (82)	37/41 (90)	0.57
**Grade ≥ 2**	9 (5)	16 (8)	0.15
**Renal toxicity**	13 (7)	13 (7)	1.00
**Reversed**	13/13 (100)	13/13 (100)	
**Grade ≥ 2**	6 (3)	5 (3)	1.00
**Hypertension Grade ≥ 2**	7 (4)	21 (11)	<0.01
**Neuropathy** (new and/or worsened from baseline)	96 (48)	22 (12)	<0.01
**Grade 1**	65 (67)	22 (100)	
**Grade 2**	24 (27)	0	
**Grade 3**	6 (6)	0	
**Reversed**	33/96 (34)	15/22 (68)	0.007

Table 3. Select pulmonary and cardiovascular events grade ≥2

Event	VRd (n=198)	KRd (n=191)	P-value
**Atrial fibrillation ≥ G2**	1 (<1%)	4* (2%)	1
**Myocardial infarction ≥ G2**	2 (1%)	1 (<1%)	1
**Pulmonary HTN G2**	0	2 (1%)	1
**Cardiac troponin I increased G3**	1 (<1%)	1* (<1%)	1
**Sinus bradycardia G2**	0	3 (2%)	1
**Pericarditis G2**	1 (<1%)	1 (<1%)	1
**Hypoxia ≥ G2**	0	2* (1%)	1
**Syncope G3**	0	1 (<1%)	1
**Dyspnea G2**	2 (1%)	1 (<1%)	1
**Pneumonitis G3**	0	1 (<1%)	1
**Chest pain G2**	0	1 (<1%)	1
**Stroke ≥ G2**	2 (1%)	0	1

*One patient had atrial fibrillation G3, hypoxia G3, and cardiac troponin I increased G3

Table 4. Multivariable analysis including maintenance therapy for all patients: PFS

	PFS	
**Variable**	Hazard Ratio (95% CI)	P value
Induction		0.035
VRd	1	
KRd	0.68 (0.47-0.97)	
Gender		>0.9
Female	1	
Male	1.01 (0.71-1.45)	
Age	1.06 (0.92-1.22)	0.4
Cardiac History		0.2
Yes	1	
No	0.75 (0.48-1.17)	
Cytogenetic risk		<0.001
Standard	1	
High	2.20 (1.52-3.17)	
R-ISS Stage		0.032
I	1	
II	1.54 (1.04-2.27)	
III	2.30 (1.09-4.86)	
Early ASCT		
No	1	
Yes	0.86 (0.58-1.27)	0.5
Maintenance therapy		
Yes	1	
No	1.15 (0.69-1.92)	0.6

Table 5A. Multivariable analysis for all patients at 5.5-year landmark point: OS

	OS	
**Variable**	Hazard Ratio (95% CI)	P value
Induction		0.063
VRd	1	
KRd	9.88 (0.88-111)	
Gender		0.7
Female	1	
Male	0.66 (0.10-4.58)	
Age	1.06 (0.92-1.22)	0.4
Cardiac History		0.8
Yes	1	
No	0.77 (0.06-10.6)	
Cytogenetic risk		>0.9
Standard	1	
High	0.89 (0.07-10.6)	
R-ISS Stage		>0.9
I	1	
II	NE	
III	NE	
Early ASCT		0.3
No	1	
Yes	4.09 (0.20-83.9)	

Table 5B. Multivariable analysis for standard-risk patients at 5.5-year landmark point: OS

Variable	OS Hazard Ratio (95% CI)	P value
Induction		0.8
VRd	1	
KRd	1.48 (0.09-25.0)	
Age	1.04 (0.88-1.23)	0.6
Early ASCT		0.8
No	1	
Yes	1.59 (0.06-40.0)	

Table 5C. Multivariable analysis for high-risk patients at 5.5-year landmark point: OS

Variable	OS Hazard Ratio (95% CI)	P value
Induction		0.044
VRd	1	
KRd	NE	
Age	1.08 (0.91-1.28)	0.4
Early ASCT		0.074
No	1	
Yes	NE	

Figure 1. Progression Free Survival of Patients Treated with VRd vs KRd with Early ASCT Censored at Time of ASCT

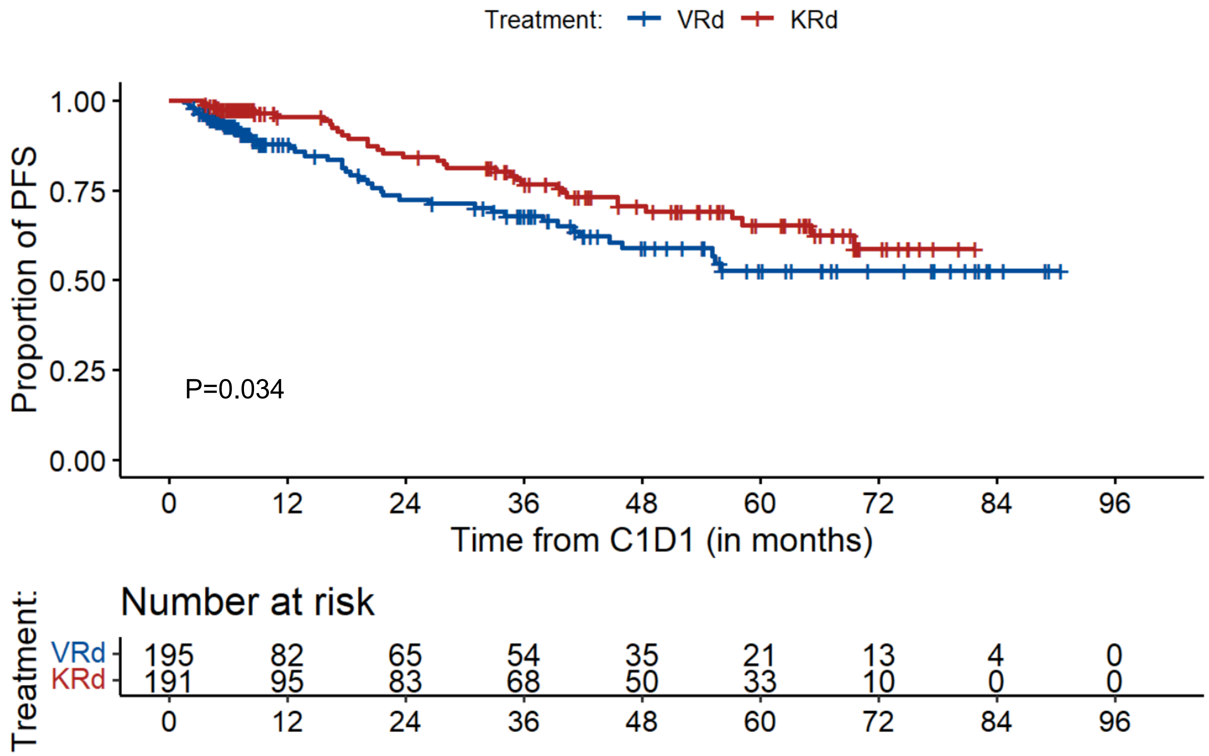

Figure 2A. OS for Patients with Follow-up Truncated at 5.5 Years

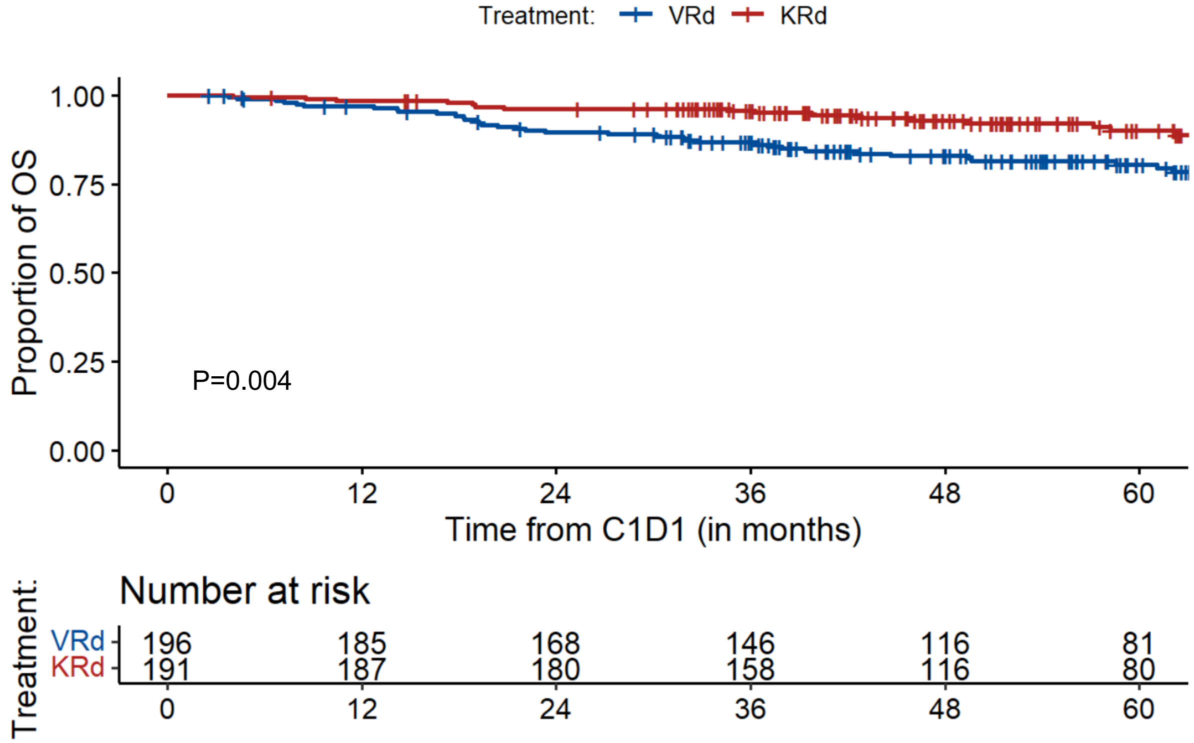

Figure 2B. OS for Patients at 5.5-year Landmark Point

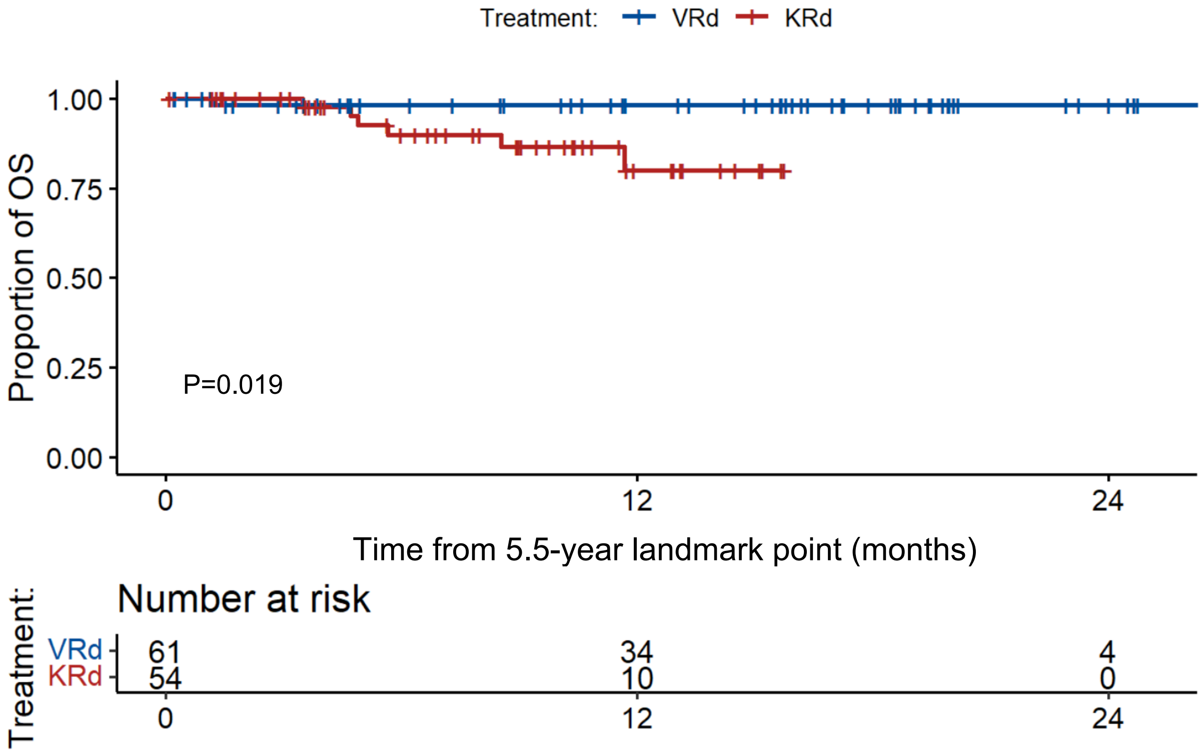

Figure 3. PFS for Patients with Gain/Amp 1q

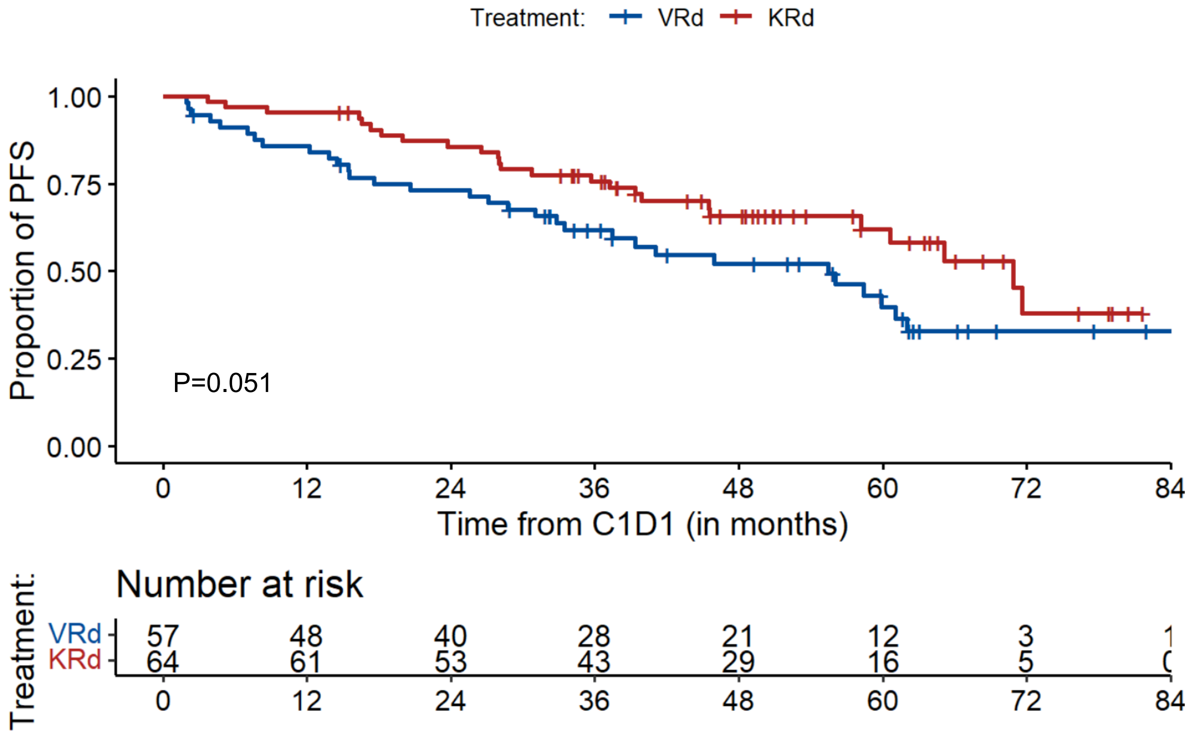

Figure 4A. EFS for Patients with Standard-risk Cytogenetics

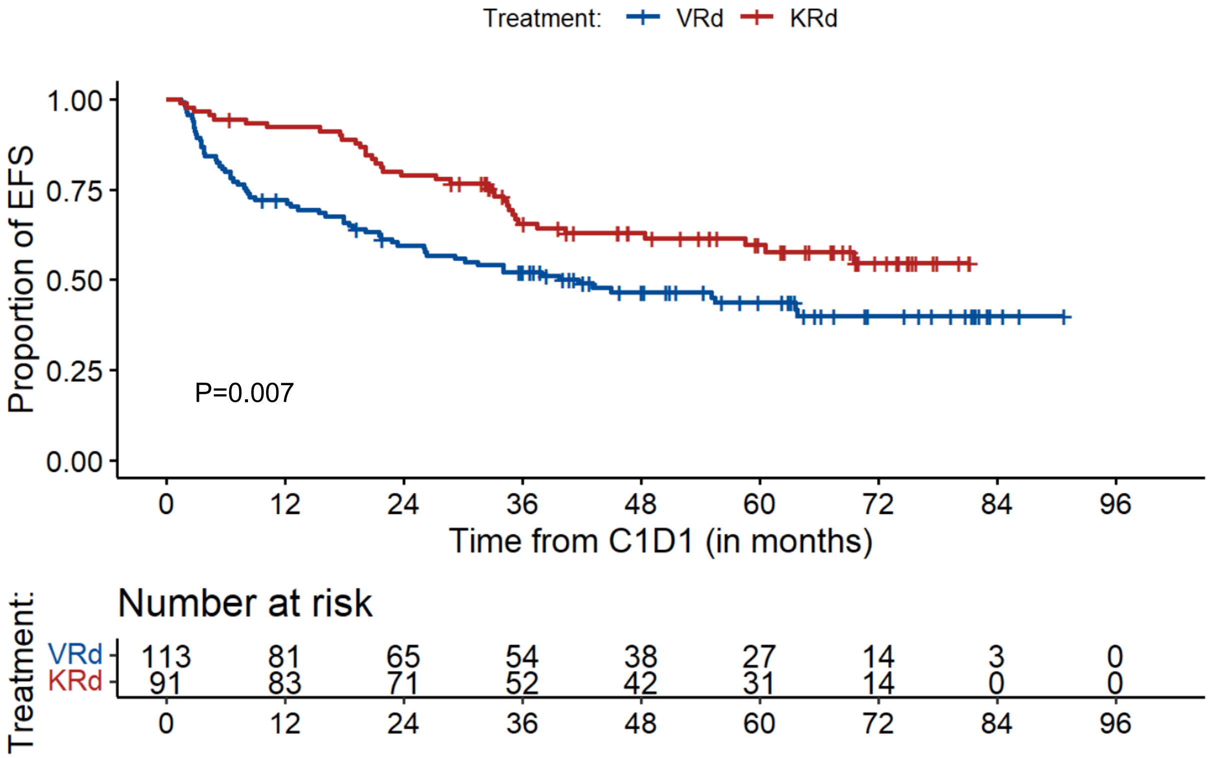

Figure 4B. EFS for Patients with High-risk Cytogenetics

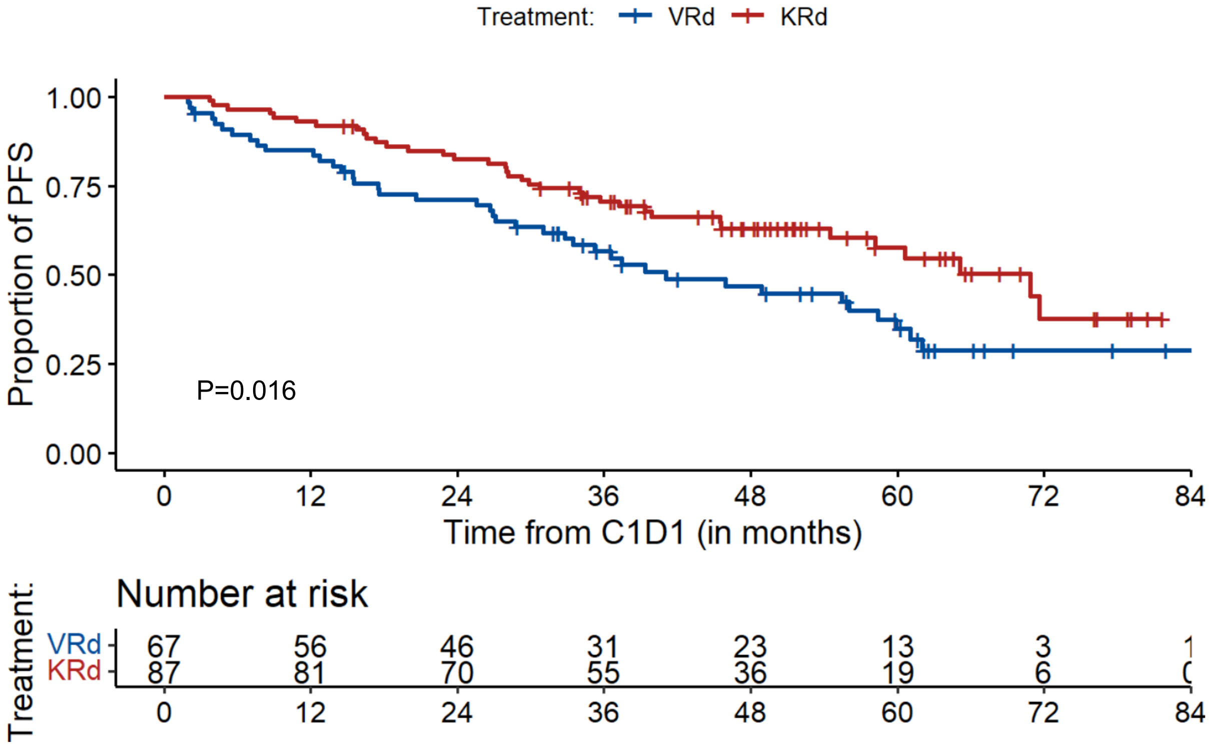

Figure 5A. OS for Patients with Standard-risk Cytogenetics with Follow-up Truncated at 5.5 Years

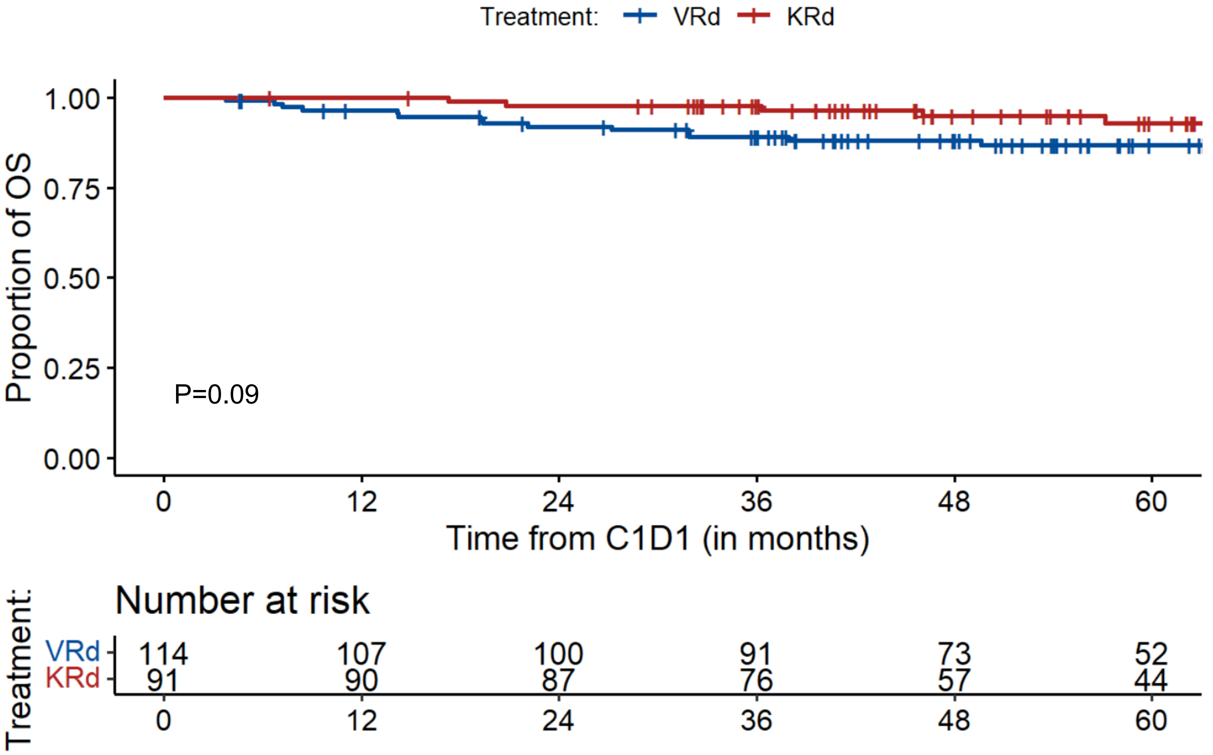

Figure 5B. OS for Patients with Standard-risk Cytogenetics at 5.5-year Landmark Point

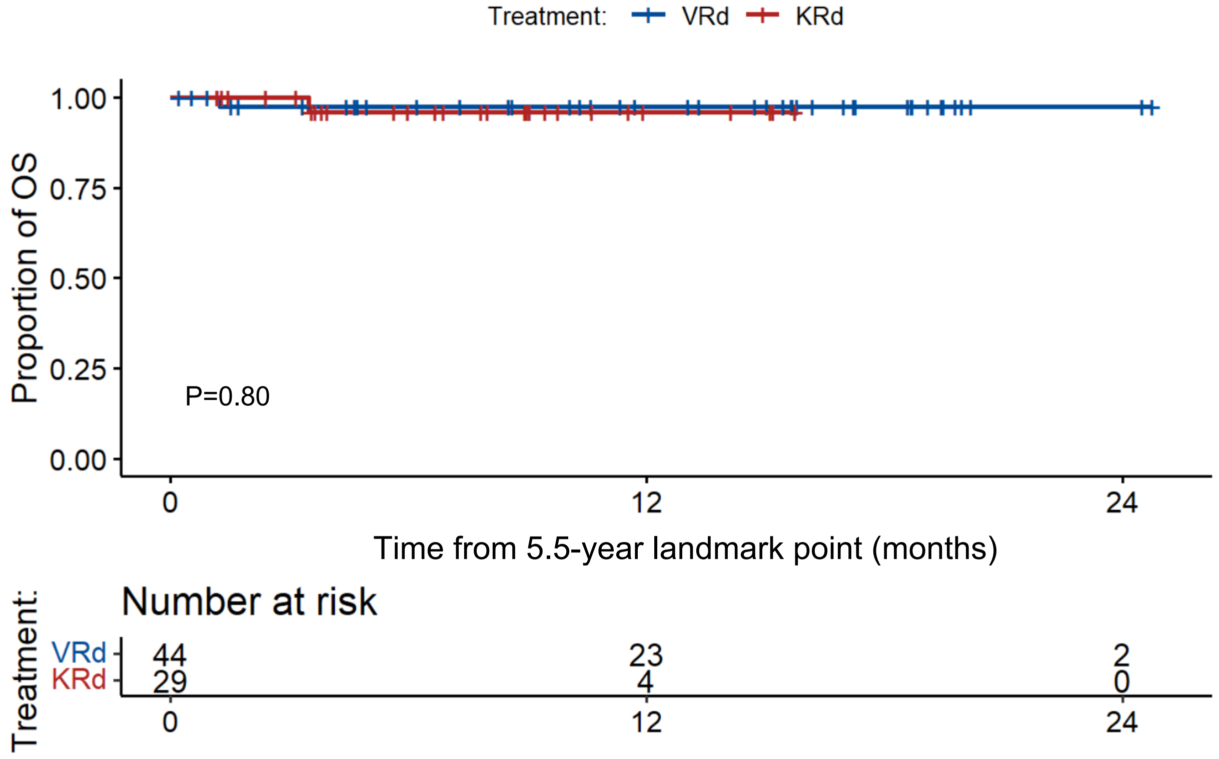

Figure 6A. OS for Patients with High-risk Cytogenetics with Follow-up Truncated at 5.5 Years

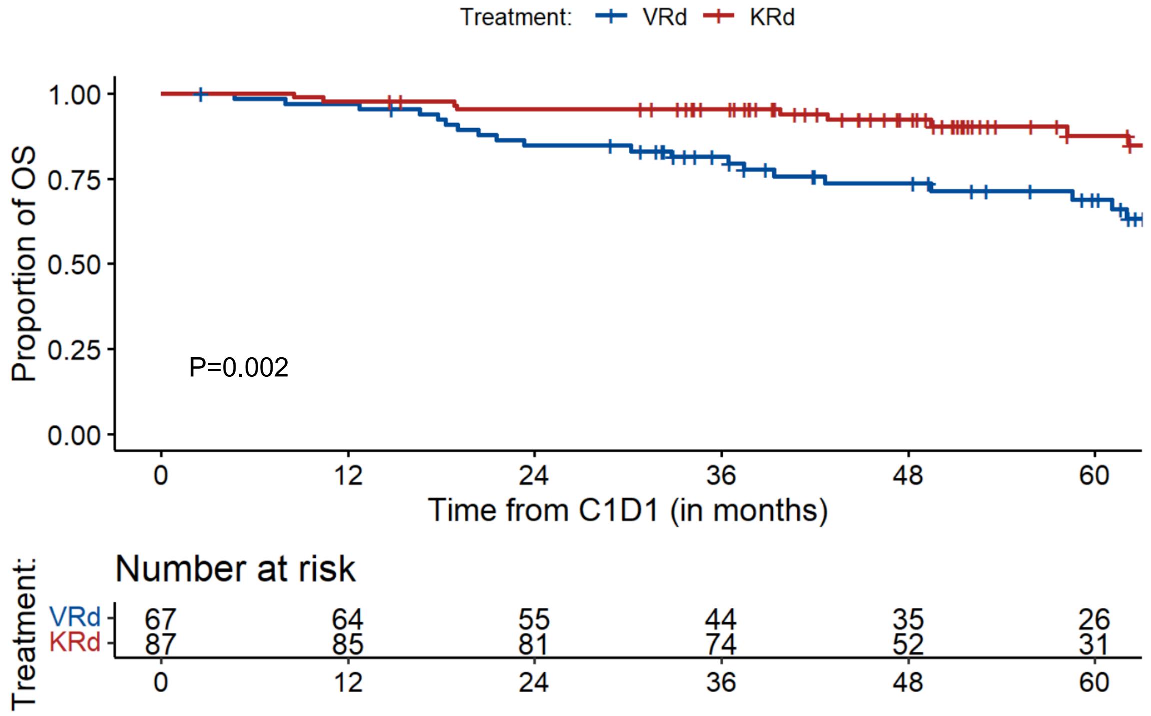

Figure 6B. OS for Patients with High-risk Cytogenetics at 5.5-year Landmark Point

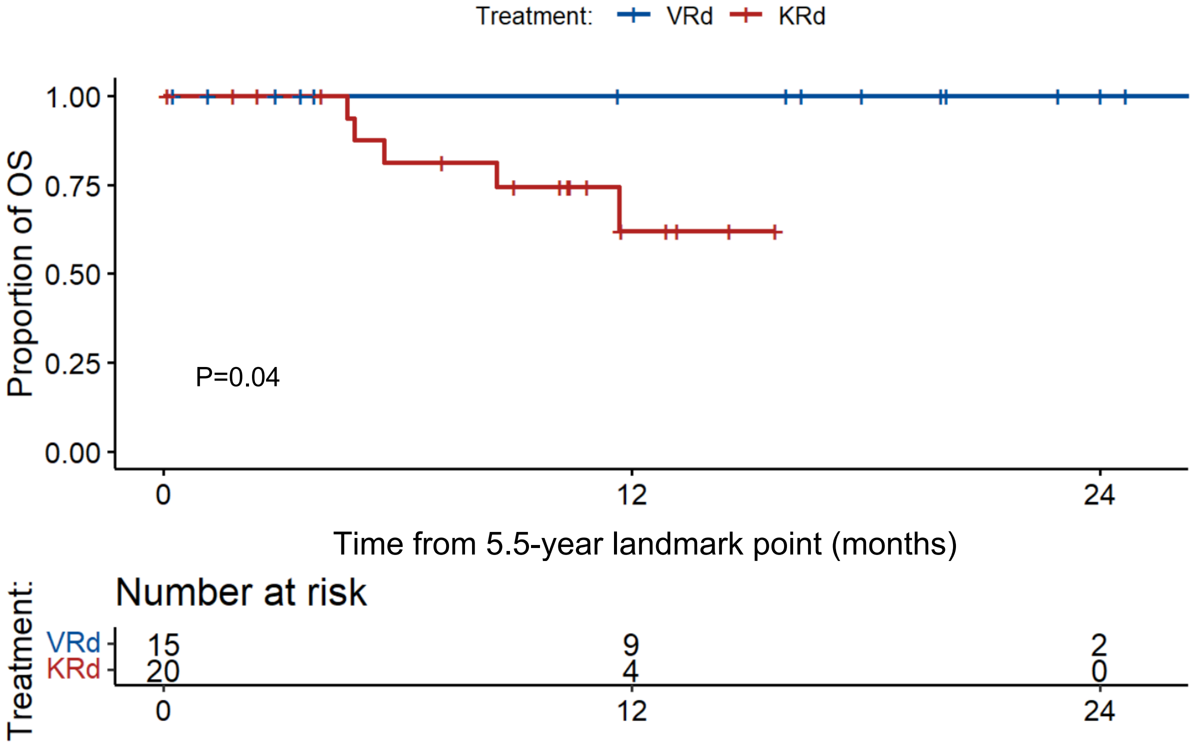

Supplement: Supplementary file 1 — Data Supplement [file 41408_2023_882_MOESM1_ESM.pdf]
